# Emotional dysregulation and attention deficit hyperactivity disorder (ADHD)

**DOI:** 10.1192/j.eurpsy.2021.579

**Published:** 2021-08-13

**Authors:** I. Ben Turkia, T. Brahim, A. Guedria, S. Bousleh, N. Gaddour

**Affiliations:** Child And Adolescent Psychiatry, Fattouma bourguiba University Hospital, Monastir, Tunisia

**Keywords:** emotional dysregulation attention-deficit/hyperactivity disorder

## Abstract

**Introduction:**

Because emotional symptoms are common in attention-deficit/hyperactivity disorder (ADHD) patients and associate with much morbidity, some consider it to be a core feature rather than an associated trait.

**Objectives:**

Assess the possibility that symptoms of emotional dysregulation should be considered as core diagnostic feature of ADHD.

**Methods:**

It’s a cross sectional study, including 60 children with ADHD and 60 children without ADHD ranging from 6 to 19 years of age (mean age 10.43 years). We defined moderate emotional dysregulation if a child had an aggregate cut-off score of ˃180 on the Anxiety/Depression, Aggression, and Attention scales of the CBCL and severe emotionaldysregulation if a child had an aggregate cut-off score of ˃ 210. This profile was selected because of its conceptual congruence with the clinical concept of emotionaldysregulation.

**Results:**

Sixty-three percent of children with ADHD had a severe emotional dysregulation versus 12% of controls (P˂0.001). Emotional dysregulation was associated with elevated rates of hyperactivity and impulsivity : Ninety-six percent of the children with hyperactivity-impulsivity, according to the Conners scale, had emotional dysregulation. With a significant correlation between emotional dysregulation and hyperactivity-impulsivity (p = 0.001). Also all children with attentional disorders exhibited emotional dysregulation and a significant correlation between emotional dysregulation and inattention has been found in both groups (p=0.000).

**Conclusions:**

Emotional dysregulation is now known to play a causal role regarding ADHD symptomatology. It should therefore be included in future theoretical models of ADHD, as well as in clinical practice when identifying the major impairments in this diagnostic group and when deciding therapeutic strategies.

